# Ophthalmic Timolol and Hospitalization for Symptomatic Bradycardia and Syncope: A Case Series

**DOI:** 10.7759/cureus.7270

**Published:** 2020-03-14

**Authors:** Syed A Abbas, Syeda M Hamadani, Umair Ahmad, Aditi Desai, Karishma Kitchloo

**Affiliations:** 1 Internal Medicine, Montefiore Medical Center/Albert Einstein College of Medicine, Bronx, USA; 2 Internal Medicine, Fatima Memorial Hospital College of Medicine and Dentistry, Lahore, PAK; 3 Internal Medicine, Ittefaq Trust Hospital, Lahore, PAK

**Keywords:** bradycardia, timolol, primary open angle glaucoma

## Abstract

Topical beta-blockers are commonly used for the management of primary open angle glaucoma (POAG). One of the rare but serious side effects of the topical beta-blockers is bradycardia, defined as a heart rate below 60 beats per minute. In few cases, the heart rate drops to much lower level resulting in syncope or symptomatic bradycardia. Topical beta-blockers are still widely used for POAG even though there are much better medications available. We present a series of four cases of symptomatic bradycardia resulting from the use of timolol eye drops and after discontinuation of the eye drops, heart rate improved to normal range (60-100 beats per minute).

## Introduction

Glaucoma is defined as an increase in intraocular pressure that can damage the optic nerve. It is the second leading cause of vision loss and its prevalence increases with age [1]. The global prevalence of the disease is estimated to be around 3.54% according to a meta-analysis report in 2014 [2]. The mainstay treatment of primary open angle glaucoma (POAG) is prostaglandin analog eye drops, but timolol, a non-selective beta-blocker, is still prescribed which used to be the first-line treatment in the 1980s for lowering intraocular pressure. The drug is used topically but can be systemically absorbed and has the potential of producing serious side effects including bronchospasm, cardiovascular, and central nervous system dysfunction [3-5]. There are a number of studies conducted which establishes association between timolol eye drops and its effect on cardiovascular functions. The cases discussed here emphasize on the importance of deprescribing topical beta-blockers especially in elderly population.

## Case presentation

Case 1

**Figure 1:**
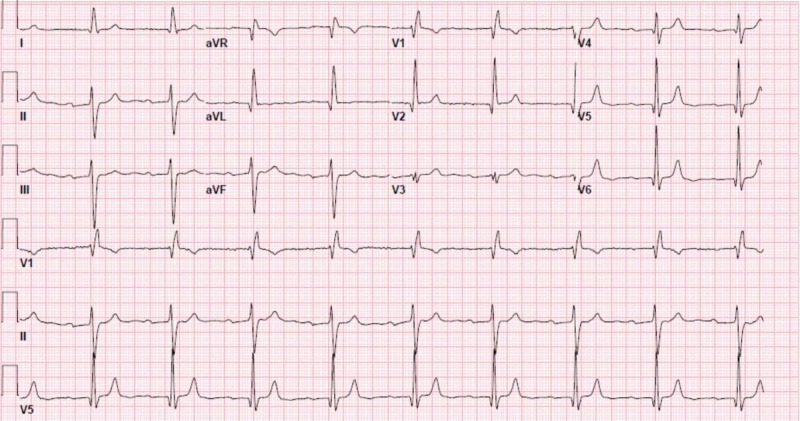
Electrocardiogram showing sinus bradycardia and first-degree atrioventricular block.

An 85-year-old man presented with a one-day history of profound weakness. He had a past medical history of coronary artery disease, moderate aortic stenosis, hypertension, chronic kidney disease, benign prostatic hyperplasia, and cataracts. His home medications included amlodipine and tamsulosin. A day prior to presentation, he received timolol eye drops at the ophthalmologist’s office. In the emergency department (ED), he was found to have a blood pressure (BP) of 70/50 mmHg and a heart rate (HR) of 49 beats/min, and rest of the physical examination was within normal limit. Electrocardiogram revealed sinus bradycardia at 55 beats/min with first-degree heart block (Figure 1). Old findings of left anterior fascicular block and right bundle branch block can also be seen. He was treated with intravenous (IV) glucagon, to which he promptly responded. Further cardiac workup did not reveal any structural heart abnormalities, and 24-hour telemetry was consistent with sinus bradycardia that improved to normal sinus rhythm after discontinuation of the ophthalmic timolol solution.

Case 2

**Figure 2:**
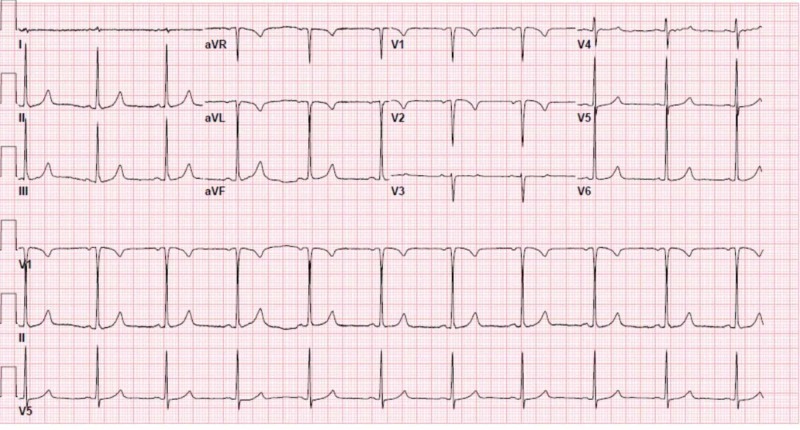
Electrocardiogram showing sinus bradycardia.

A 75-year-old female with past medical history of hypertension, hyperlipidemia, mild dementia, and glaucoma presented with complaint of generalized weakness. Initial BP was 59/33 mmHg, HR 61 beats/min, and finger stick glucose of 93 mg/dl. She was alert, oriented, and no neurological deficit was noted on the physical exam. Basic laboratory workup was unremarkable, and electrocardiogram (EKG) showed sinus bradycardia (Figure 2). Home lisinopril was discontinued, and the patient was given gentle hydration resulting in resolution of symptoms. The patient was discharged home in a medically stable condition. Four days later, she again presented to the ED with similar complaints. At the time of this presentation, her vitals were BP 95/66 mmHg and HR 60 beats/min. Again the blood work and EKG were non-contributory. When asked, the patient and daughter reported onset of symptoms sometimes after instillation of timolol eye drops. Eye drops were held and the patient was discharged home in medically stable condition after normalization of vitals. Follow-up call was done after five days, and the patient reported no recurrence of symptoms with normalization of BP and HR.

Case 3

**Figure 3:**
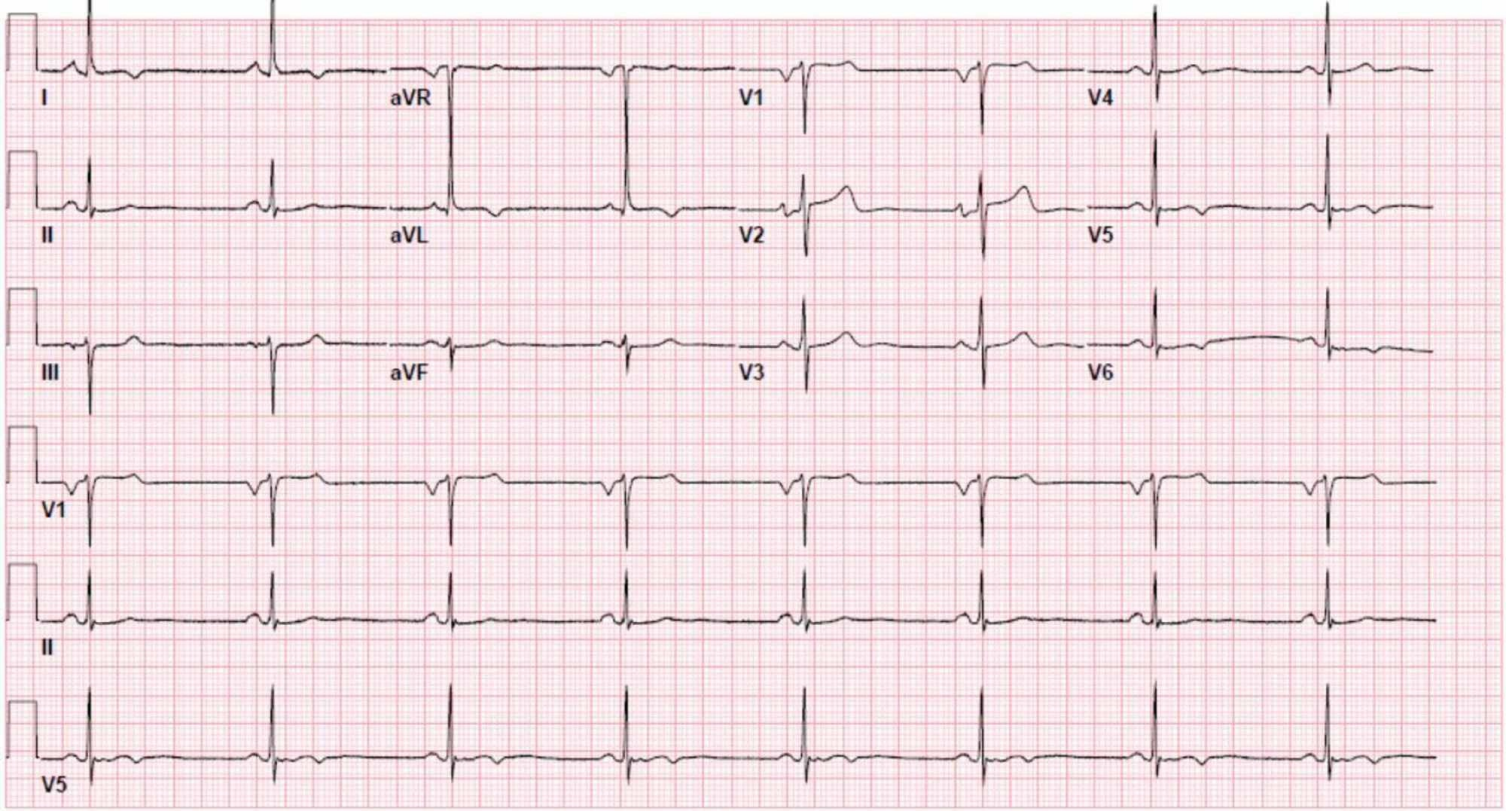
Electrocardiogram showing sinus bradycardia.

An 88-year-old female with past medical history of hypertension, hyperlipidemia, glaucoma, and vertigo presented to ED with complaints of lightheadedness, palpitations, and near syncope. Initial vital signs showed BP of 110/70 mmHg and heart rate of 47 beats/min (Figure 3). Telemonitoring was significant for sinus bradycardia. Timolol was held in view of bradycardia, and the patient did not report further episodes of lightheadedness.

Case 4

**Figure 4:**
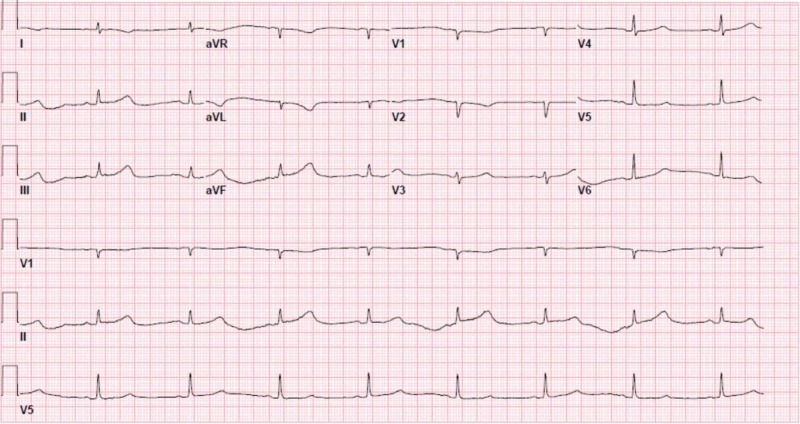
Electrocardiogram showing sinus bradycardia.

A 69-year-old female presented to the ED with dizziness for four days and a syncopal episode. In the ED, she was bradycardic (45-50 beats/min) with a BP of 146/73 mmHg (Figure 4). Her orthostatic vitals were negative. Physical examination, including a detailed neurological exam, was unremarkable. She received a dose of meclizine in the ED, which showed some improvement in her symptoms but the bradycardia persisted. Her syncope workup was negative, and all cardiogenic andneurogenic causes were ruled out. Further investigation and review of medications revealed timolol eye drops. Holding the eye drops showed significant improvement in her heart rate. Her eye drops were stopped on discharge, and she reported no dizziness episodes on the outpatient follow-up visit.

## Discussion

The estimated prevalence of glaucoma in the US population, aged more than 40 years, is around 2.1% (approximately 2.9 million individuals) [6]. Approximately 90% of glaucoma are open angle glaucoma for which topical ophthalmic solutions are prescribed. With the recent advancement in medicine, prostaglandin analog ophthalmic solution is the first-line treatment for POAG. However, before the introduction of prostaglandin analogs, beta-blocker eye drops were the mainstay of treatment.

Timolol, a non-selective beta-blocker, ophthalmic solutions are well known for the management of this condition which is also associated with some serious cardiovascular side effects including bradycardia. The systemic bioavailability, pharmacokinetics, and cardiopulmonary effects of 0.5% timolol ophthalmic solution are comparable to IV timolol [7]. The systemic bioavailability of ophthalmic timolol is around 78% compared with oral timolol which is around 61% due to first pass metabolism, and also the drug was detectable in plasma after 15 minutes of administering the ophthalmic solution [7]. Regarding the pharmacokinetics of the 0.5% ophthalmic timolol, one drop to each eye is estimated to be around 10 mg oral dose [8,9].The half-life of the drug is around four to five hours, and it is metabolized by cytochrome P450 2D6 enzyme (CYP2D6) to inactive metabolites which are excreted by the kidneys [10,11].

Although POAG is more common in elderly population, precautions should be taken in prescribing ophthalmic timolol as the majority of these patients have underlying cardiovascular problems.

The duration of action of the ophthalmic solution is more pronounced and also is more potent in elderly patients as compared to younger ones [12]. There have been reported cases of bradycardia, variable degrees of atrioventricular block, and complete heart blocks with the use of ophthalmic timolol [13-15]. To avoid systemic side effects of the eye drops, gel formulations were developed but the side effects related to beta-blockade of the receptors were similar in both the groups [16,17].

Even though the first-line treatment for POAG is prostaglandin analogs, we still have many patients in internal medicine clinics and wards who are prescribed non-selective beta-blockers resulting in serious adverse effects. Hence, the clinicians must be aware of the possibility and should consider deprescribing the topical beta-blocker eye drops. Preference should be given to prostaglandin analogs, especially in elderly patients.

## Conclusions

We presented a case series of ophthalmic timolol-induced bradycardia resulting in multiple hospital admission. After ruling out any underlying pathology, the eye drops were discontinued and patients were followed up which showed heart rate improving to normal range. The primary purpose of this case series is to emphasize the side effect profile of non-selective beta-blocker eye drops in elderly population and also change of practice to prescribing prostaglandin analog eye drops, which is now the first-line treatment for POAG.
